# *C. elegans* midbodies are released, phagocytosed and undergo LC3-dependent degradation independent of macroautophagy

**DOI:** 10.1242/jcs.190223

**Published:** 2016-10-15

**Authors:** Gholamreza Fazeli, Michaela Trinkwalder, Linda Irmisch, Ann Marie Wehman

**Affiliations:** Rudolf Virchow Center for Experimental Biomedicine, University of Würzburg, Würzburg 97080, Germany

**Keywords:** Abscission, Cell division, Macroautophagy, Midbody, Phagocytosis

## Abstract

In animals, the midbody coordinates the end of cytokinesis when daughter cells separate through abscission. The midbody was thought to be sequestered by macroautophagy, but recent evidence suggests that midbodies are primarily released and phagocytosed. It was unknown, however, whether autophagy proteins play a role in midbody phagosome degradation. Using a protein degradation assay, we show that midbodies are released in *Caenorhabditis* *elegans*. Released midbodies are known to be internalized by actin-driven phagocytosis, which we show requires the RAB-5 GTPase to localize the class III phosphoinositide 3-kinase (PI3K) complex at the cortex. Autophagy-associated proteins, including the Beclin 1 homolog BEC-1 and the Atg8/LC3-family members LGG-1 and LGG-2, localize around the midbody phagosome and are required for midbody degradation. In contrast, proteins required specifically for macroautophagy, such as UNC-51 and EPG-8 (homologous to ULK1/Atg1 and Atg14, respectively) are not required for midbody degradation. These data suggest that the *C. elegans* midbody is degraded by LC3-associated phagocytosis (LAP), not macroautophagy. Our findings reconcile the two prevailing models on the role of phagocytic and autophagy proteins, establishing a new non-canonical role for autophagy proteins in midbody degradation.

## Abstract (precis)

**Summary:** Autophagy proteins are required for the degradation of midbodies. In *C. elegans*, Atg8/LC3-family proteins act during phagosome maturation rather than during macroautophagy.

## INTRODUCTION

Cytokinesis is the final step of cell division through which daughter cells become physically separated (reviewed in Green et al., 2012). In response to signaling from the anaphase spindle, an actomyosin ring contracts to create an intercellular bridge. The spindle midzone meets the contractile ring to form the midbody, whose proteins coordinate the final separation of the daughter cells through abscission. Abscission requires concerted control of vesicular trafficking as well as the actin, microtubule and septin cytoskeletons (Schiel et al., 2013). Considering the potent signaling roles of midbody proteins during cytokinesis, the cell needs to exert highly regulated programs to resolve the midbody at and after abscission. The fate of the post-mitotic midbody remnant (hereafter referred to simply as the midbody) is also unclear, despite reports stating that its positioning influences cell polarity and cell fate (Kuo et al., 2011; Morais-de-Sa and Sunkel, 2013; Singh and Pohl, 2014).

Two distinct models are currently accepted for the mechanisms of midbody sequestration: macroautophagy or release (Schiel et al., 2013). In the autophagy model, asymmetric abscission on one side of the midbody leads to intercellular bridge retraction and the cytoplasmic inheritance of the midbody by one daughter cell. The cytoplasmic midbody is then sequestered in a double-membrane autophagosome. The autophagy model is based on the observation that proteins of the Atg8/LC3 family, which are typically used as markers for autophagosomes, localize around the internalized midbody (Pohl and Jentsch, 2009; Kuo et al., 2011). The lipidation of Atg8/LC3 proteins driven by ATG7 is also required for midbody degradation, in addition to other proteins associated with autophagy, such as the class III phosphoinositide 3-kinase (PI3K) VPS34 and its regulatory subunit Beclin 1 (Pohl and Jentsch, 2009; Kuo et al., 2011; Isakson et al., 2013). However, one puzzling aspect of the autophagy model is that it would leave the signaling proteins of the spindle midzone and actomyosin ring unchecked in the cytoplasm until autophagic engulfment occurs, potentially endangering the cell through misregulation of the cytoskeleton or vesicular trafficking.

As an alternative to the autophagy model, it has been observed that the midbody is released outside the cell by symmetric abscission (Dubreuil et al., 2007; Ettinger et al., 2011). Midbodies from many types of mammalian cells are released *in vitro* (Crowell et al., 2014) and murine neural progenitor midbodies are released into the cerebrospinal fluid *in vivo* (Arai et al., 2015). Release efficiently sequesters midbody proteins where they can be safely internalized by receptor-mediated phagocytosis by daughter cells or even by neighboring cells (Chai et al., 2012; Ou et al., 2014; Crowell et al., 2014). Indeed, neighboring cells take up midbodies *in vivo,* such as somatic cyst cells internalizing germ cell midbodies in *Drosophila* (Salzmann et al., 2014).

Despite the apparent conflicts in the autophagy and phagocytosis models, no one has tested whether these models actually correspond to a single pathway, specifically whether autophagy proteins are required for degradation of the midbody phagosome by the lysosome. This question is especially pertinent given the discovery of noncanonical autophagy pathways such as LC3-associated phagocytosis (LAP), in which autophagy proteins are required for the degradation of phagocytosed bacteria or phagocytosed cell corpses independent of macroautophagy. Core autophagy proteins involved in LAP include Atg8/LC3-family proteins, ATG7, PI3K and Beclin 1 (Sanjuan et al., 2007; Florey et al., 2011; Martinez et al., 2011). Association of Atg8/LC3 proteins with the phagosome membrane during LAP is important for phagosome–lysosome fusion and degradation of the phagosomal cargo. It has even been proposed that the midbody could be degraded by LAP, but the authors observed LC3 staining on midbodies infrequently in cultured cells (Crowell et al., 2014), in contrast to previous reports (Pohl and Jentsch, 2009; Kuo et al., 2011). Thus, the symmetry of abscission and the fate of the midbody remain unclear.

In this study, we use time-lapse imaging to elucidate the fate of the midbody after cytokinesis in *Caenorhabditis elegans* embryos. The *C. elegans* early embryo is an ideal model system to determine the role of autophagy proteins in midbody degradation, because midbodies are phagocytosed with a stereotypical timing and an asymmetric pattern of inheritance (Ou et al., 2014; Singh and Pohl, 2014). Using a degradation tag, we demonstrate that midbodies are released outside of cells even when they are internalized asymmetrically by a daughter cell. Phagocytosis depends on signaling pathways mediated by the RAC1 homolog CED-10 (Chai et al., 2012; Ou et al., 2014). We show that the class III PI3K complex and the small GTPase RAB-5 are required to localize the phagocytic receptor CED-1 (homologous to MEGF11) to the plasma membrane. We further reveal that proteins required for both LAP and autophagy, such as PI3K subunits and Atg8/LC3 proteins, decorate midbody phagosomes and are required for their degradation. In contrast, we find proteins specifically required for autophagy, such as UNC-51 and EPG-8 (homologous to ULK1 and ATG14, respectively) (Martinez et al., 2011, 2015; Yang and Zhang, 2011), are not required for degradation of the midbody phagosome. Taken together, our results demonstrate that after midbody release by symmetric abscission, LAP is required for the intracellular degradation of midbody remnants.

## RESULTS

### PI3K and lipidated Atg8/LC3 proteins are required for midbody degradation

**Fig. 1.**
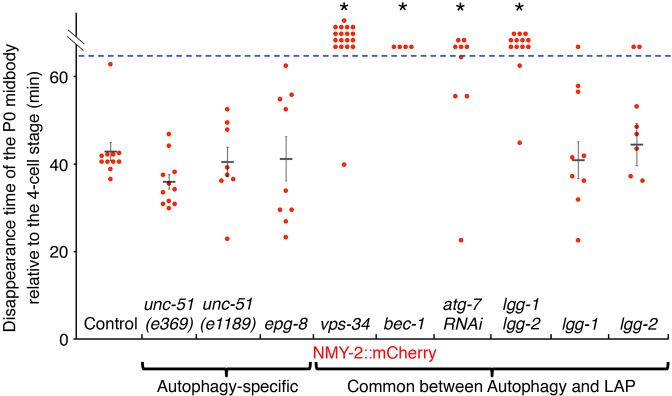
**A subset of autophagy-related genes is required for midbody degradation.** In embryos treated with *atg-7* RNAi (*n*=9 embryos), *lgg-1; lgg-2* double mutants (*n*=13), and *bec-1* (*n*=4) or *vps-34* (*n*=18) maternal-zygotic mutants. NMY-2::mCh persists longer than in controls (*n*=11, *P<0.01). The disappearance time in *lgg-1* (*n*=9) or *lgg-2* (*n*=8) single mutants, as well as in *unc-51* (*n*=11 and 8 for *e369* and *e1189*, respectively) or *epg-8* maternal-zygotic mutants (*n*=9), was not significantly different than controls (*P*>0.05). Each point represents a single embryo and the mean±s.e.m. is also shown. Student's *t*-test with Bonferroni correction was used for statistical analysis.

Given that midbodies persist in autophagy mutants in cultured mammalian cells (Pohl and Jentsch, 2009; Kuo et al., 2011; Isakson et al., 2013), we tested whether autophagy proteins are required for the degradation of phagocytosed midbodies in *C. elegans* embryos. We first analyzed the two *C. elegans* members of the Atg8/LC3 family, LGG-1 (part of the GABARAP/GATE16 subfamily) and LGG-2 (part of the MAP1LC3 subfamily). LGG-1 and LGG-2 are incorporated into and are required for elongation of the autophagosome membrane, similar to yeast Atg8 (Manil-Segalen et al., 2014). We labeled midbodies using an mCherry-tagged NMY-2 (non-muscle myosin; NMY-2::mCh) reporter to label the actomyosin midbody ring and midbody remnants (Shelton et al., 1999). In control embryos, NMY-2::mCh disappeared from the first embryonic midbody (P0 midbody) at 43±7 min after the four-cell stage (mean±s.d., *n*=7, Fig. 1, see also Fig. 6C). Surprisingly, in *lgg-1; lgg-2* double mutants, fluorescence remained constant on the internalized midbody (*n*=6, Fig. 1, see also Fig. 6C), demonstrating that LGG-1 and LGG-2 are required for degradation of the midbody phagosome. LGG-1 and LGG-2 act redundantly, because mCherry-tagged midbodies in *lgg-1* or *lgg-2* single mutants lost fluorescence and disappeared (Fig. 1). LGG-1 and LGG-2 must also be conjugated with the lipid phosphatidylethanolamine, because the NMY-2::mCh reporter also persisted when *atg-7* was depleted using RNAi (Fig. 1). The E1-like activating enzyme ATG-7 is required for the lipidation of LGG-1 and LGG-2 (Tian et al., 2010). These data reveal that both the LC3 and GABARAP/GATE16 subfamilies are required for degradation of the midbody phagosome after being lipidated.

We next analyzed the class III PI3K complex, which is involved in an earlier step of macroautophagy, namely the initiation of the phagophore isolation membrane. PI3K is comprised of the kinase VPS-34 and modulatory subunits such as the Beclin 1 homolog BEC-1 (Funderburk et al., 2010). In PI3K-deficient *vps-34* or *bec-1* maternal-zygotic mutants (i.e. embryos lacking both maternal and embryonic protein), we also observed that NMY-2::mCh failed to disappear from the P0 midbody. These data show that proteins classically associated with autophagy are required for the degradation of phagocytosed midbodies, which was not predicted by either of the current midbody degradation models.

### Before phagocytosis, midbodies are released outside cells

Given this unexpected role for autophagy proteins, we wanted to confirm that midbodies are released in the early embryo. The P0 midbody forms when the zygote (P0) divides to an anterior (AB) and a posterior (P1) blastomere. The P0 midbody is then phagocytosed by a daughter cell (Green et al., 2013; Ou et al., 2014; Singh and Pohl, 2014). Before phagocytosis, the midbody could still be attached to one daughter cell (after asymmetric abscission), which could require autophagy proteins to seal the phagosome through phagophore elongation (Ettinger et al., 2011). Alternatively, the midbody could be released into the extracellular space by abscission on both sides of the midbody. To discover whether the P0 midbody is released, we used the ZF1-mediated degradation technique (Nance et al., 2003). The first CCCH finger (ZF1) of PIE-1 targets proteins for proteasome-mediated degradation in somatic cells (Fig. S1A) (Reese et al., 2000), but released extracellular vesicles are protected from ZF1 degradation (Wehman et al., 2011). Thus, ZF1-tagged reporters can track extracellular structures and reveal connections to the cytoplasm.

We chose to tag NMY-2 with the ZF1 domain, because midbody ring components are located more superficially than proteins of the centralspindlin complex in the core of the midbody (Green et al., 2012). We first verified that the ZF1 tag did not disrupt midbody phagocytosis and analyzed internalization of the first three midbodies using a ZF1-tagged NMY-2::GFP reporter. To better judge internalization, the strain also expressed a plasma membrane reporter using the pleckstrin homology (PH) domain of PLCδ1, which binds phosphatidylinositol 4,5-bisphosphate (PIP_2_) (Audhya et al., 2005). NMY-2::GFP::ZF1 localized to contracting midbody rings and persisted in the midbody remnant (Movie 1), similar to other NMY-2 reporters (Green et al., 2013). Similar to reports with other midbody markers (Ou et al., 2014; Singh and Pohl, 2014), we observed stereotypical internalization of the first three midbodies in NMY-2::GFP::ZF1 reporter embryos (Table 1), with daughter cells internalizing the P0 midbody and the P1 midbody (formed when the posterior P1 cell divides), whereas a neighboring cell internalizes the AB midbody (formed when the anterior AB cell divides). Thus, the ZF1 tag does not disrupt midbody phagocytosis.

**Table 1.**
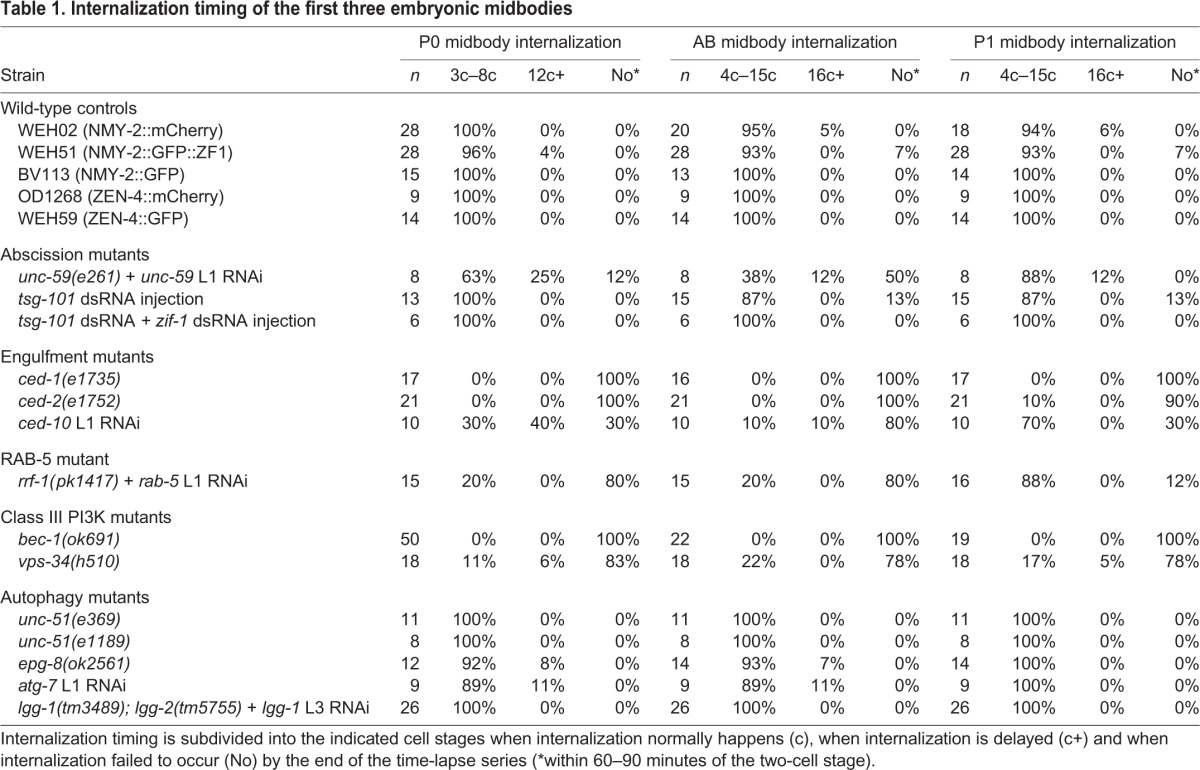
**Internalization timing of the first three embryonic midbodies**

**Fig. 2.**
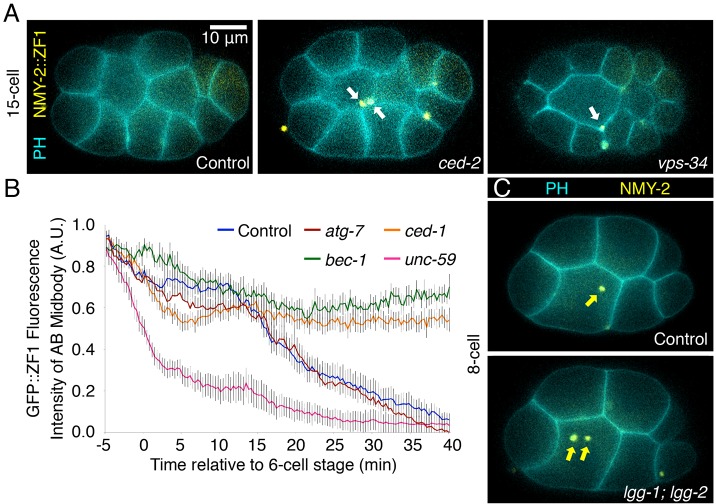
**Midbodies are released extracellularly and macroautophagy is not required for midbody internalization.** (A) In control WEH51 embryos expressing mCh::PH_PLCδ1_ on membranes (PH, cyan), midbodies have lost NMY-2::GFP::ZF1 (yellow) fluorescence by the 15-cell stage (*n*=22/25 embryos). See also Movie 1. Midbody fluorescence (arrows) is retained in *ced-2* and *vps-34* mutants (*n*=12/12 and 11/13, respectively). Only one *z*-slice is shown. (B) Control midbodies (*n*=12), as well as midbodies from autophagy-deficient embryos treated with *atg-7* RNAi (*n*=10), start losing NMY-2::GFP::ZF1 fluorescence on the AB midbody by 20 min after the six-cell stage, whereas the abscission mutant *unc-59* lost fluorescence earlier (*n*=7, *P*<0.01). Midbodies that were not phagocytosed maintained fluorescence for at least 40 min after the six-cell stage (*n*=6 and 11 for *bec-1* and *ced-1*, respectively). Results are mean±s.e.m. See also Fig. S1. (C) Midbodies internalize (arrows) in *lgg-1; lgg-2* maternal-zygotic mutants (*n*=26) as in control embryos (*n*=28) expressing NMY-2::mCh (yellow) and GFP::PH_PLCδ1_ (cyan). Student's *t*-test with Bonferroni correction was used for statistical analysis.

To determine whether the NMY-2::GFP::ZF1 reporter could be degraded from the midbody, we first measured midbody fluorescence in embryos expressing NMY-2::GFP::ZF1 in known abscission mutants. Septins and the ESCRT machinery act during late stages of abscission after the midbody ring has closed around the central spindle (Green et al., 2012). It has been previously observed that abscission defects in embryos treated with RNAi for the septin UNC-59 or the ESCRT-I component TSG-101 lead to defects in internalization of the midbody remnant (Green et al., 2013). AB cells begin ZF1 degradation ∼5 min before the six-cell stage. As predicted if midbodies are still attached to AB cells, NMY-2::GFP::ZF1 underwent rapid degradation on the AB midbody in *unc-59* abscission mutants (Fig. 2B) as well as in embryos treated with *tsg-101* RNAi (Fig. S1B). ZF1 degradation occurred in abscission mutants even when the internalization defects were relatively mild (Table 1), thus proving a sensitive assay for abscission defects. These results confirm that NMY-2::GFP::ZF1 on the outer layer of the midbody is degradable. We next confirmed that midbody-associated NMY-2::GFP::ZF1 is degraded through the normal ZIF-1-mediated pathway. ZIF-1 is the substrate recognition factor for the cullin E3 ubiquitin ligase that targets ZF1-containing proteins for subsequent proteasome-mediated destruction (DeRenzo et al., 2003). NMY-2::GFP::ZF1 fluorescence did not undergo rapid degradation in *tsg-101 zif-1* double RNAi-treated embryos (Fig. S1B), demonstrating that the ZF1 reporter undergoes degradation by the expected ZIF-1-dependent pathway. Thus, NMY-2::GFP::ZF1 fluorescence can be used to probe whether midbodies are connected to the cytoplasm.

To determine whether midbodies are released before phagocytosis, we first analyzed the AB midbody in control embryos. It was expected that the AB midbody would be released, because a neighboring non-daughter cell normally internalizes it (95%, *n*=43) (Ou et al., 2014; Singh and Pohl, 2014). AB cells began degrading NMY-2::GFP::ZF1 ∼11 min before AB midbody internalization (∼6 min after the six-cell stage). In control embryos, fluorescence was steady on the AB midbody while it was at the cell surface and for several minutes after internalization (Fig. 2B, *n*=11). Thus, in contrast to abscission mutants, the AB midbody is normally protected from ZF1 degradation while at the cell surface due to extracellular release. Fluorescence later decreased until the NMY-2::GFP::ZF1 reporter disappeared by the 15-cell stage (28±6 min after internalization; mean±s.d.) (Fig. 2A,B; see also Fig. 6A, *n*=11). This suggests that the AB midbody is also protected for a period after internalization, likely because it is inaccessible to the cytoplasm inside the phagosome. In support of this, we observed the PIP_2_ reporter surrounding midbodies after internalization, confirming that midbodies are wrapped in a plasma membrane-derived vesicle (Fig. S1D).

To ensure that midbodies were accessible to ZF1-mediated degradation for a sufficiently long time to demonstrate they are released, we also tested whether NMY-2::GFP::ZF1 would still be protected from degradation when midbodies were not phagocytosed. The MEGF11 homolog CED-1 and the CRKL homolog CED-2 belong to two receptor-mediated signaling pathways required for midbody internalization in *C. elegans* embryos (Chai et al., 2012; Ou et al., 2014). Both pathways activate the RAC1 homolog CED-10 to rearrange the actin cytoskeleton and induce phagocytic cup formation, resulting in engulfment of cell corpses and midbodies (Chai et al., 2012). In *ced-1* (*n*=9) and *ced-2* (*n*=8) engulfment mutants, NMY-2::GFP::ZF1 fluorescence remained constant on the AB midbody (Fig. 2A,B), demonstrating that the midbody has no cytoplasmic connection to an AB cell. Thus, as predicted, the AB midbody is released and the ZF1 technique can be used to determine whether other midbodies are released.

We next examined the ZF1 reporter on the P0 midbody, which is normally internalized by a daughter cell. We found that P0 midbodies in phagocytic mutants also had constant NMY-2::GFP::ZF1 fluorescence (Fig. 2A; Fig. S1C), demonstrating that midbodies asymmetrically engulfed by daughter cells are protected from ZF1 degradation like midbodies asymmetrically engulfed by neighboring cells. Midbodies were also found on the outer surface of *ced-2* embryos (*n*=9/12) where they moved independently, indicating that they were no longer connected to a daughter cell. Thus, midbodies are released after abscission cuts on both sides of the midbody, even when internalized asymmetrically by a daughter cell.

### Midbodies are not sequestered or internalized through macroautophagy

Despite the fact that the ZF1 data supported the release and phagocytosis model, we also tested whether macroautophagy is required to protect the midbody from ZF1-mediated degradation in the cytoplasm. If phagophore elongation was needed to sequester a cytoplasmic midbody or to seal the midbody phagosome, we predicted we would see a rapid drop in ZF1 reporter fluorescence, as observed in abscission mutants. We measured NMY-2::GFP::ZF1 fluorescence in embryos treated with *atg-7* RNAi and observed a pattern similar to control embryos (Fig. 2B). There was no rapid loss of fluorescence in *atg-7* knockdowns when ZF1 degradation began in the AB cytoplasm, as was seen in abscission mutants (Fig. 2B). This demonstrates that lipidated LGG-1 and LGG-2 are not required for sequestration of the midbody from the cytoplasm through phagophore elongation. Thus, midbodies are released and autophagy proteins are not needed to seal the midbody phagosome.

We next investigated whether Atg8/LC3 and PI3K proteins are required for midbody internalization. In control embryos, the first midbody is internalized by the eight-cell stage (Fig. 2C, Table 1). Double mutants lacking both LGG-1 and LGG-2 showed normal midbody internalization (Fig. 2C, Table 1), further indicating that phagophore elongation does not play a role in phagocytosis. Similarly, midbody internalization was normal when we used *atg-7* RNAi to disrupt LGG-1 and LGG-2 lipidation (Table 1). Thus, midbody sequestration by macroautophagy does not play a role in midbody internalization.

In contrast to *atg-7* and the Atg8/LC3 homologs, midbodies did not internalize in PI3K-deficient *vps-34* or *bec-1* maternal-zygotic mutants (Fig. 2A, Table 1). The internalization phenotype was similar to *ced* engulfment mutants (Table 1) (Chai et al., 2012; Ou et al., 2014) and likely explains why the NMY-2::mCh fluorescence did not disappear (Fig. 1). PI3K phosphorylates phosphatidylinositol and is required for abscission and phagocytosis in mammals in addition to autophagy (reviewed in Levine et al., 2015). Thus, PI3K could act during abscission or phagocytosis to internalize *C. elegans* midbodies.

### PI3K is not required for abscission in *C. elegans*

To determine whether PI3K is required for abscission in *C. elegans* embryos, we used the ZF1 technique to test whether midbodies are still released in PI3K mutants. In contrast to abscission mutants, NMY-2::GFP::ZF1 fluorescence remained steady on the midbody in *bec-1* and *vps-34* maternal-zygotic mutants (Fig. 2A,B). This finding reveals that midbodies are not connected to the cytoplasm in PI3K mutants, similar to our observations in engulfment mutants. We also did not observe cytokinesis failure or multinuclear cells in *bec-1* and *vps-34* mutants. Thus, surprisingly, PI3K does not appear to be required for abscission in *C. elegans* in contrast to other systems (Levine et al., 2015).

### PI3K is required for midbody phagocytosis

**Fig. 3.**
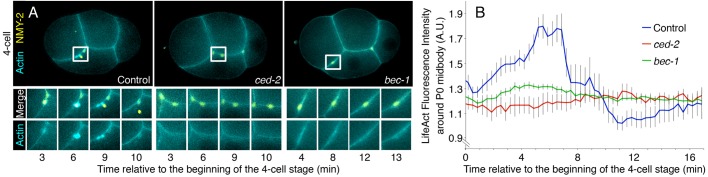
**PI3K acts during phagocytosis to internalize the midbody.** (A) In control BV113 embryos (left, *n*=13 embryos), actin accumulates around the P0 midbody before internalization. Actin does not accumulate in *ced-2* (*n*=11) or *bec-1* (*n*=5) mutants. NMY-2::GFP labels midbodies (yellow) and LifeAct::RFP labels F-actin (cyan). See also Fig. S2 and Movies 2–4. (B) LifeAct::RFP fluorescence intensity is significantly reduced around the P0 midbody in *ced-2* (red) and *bec-1* (green) mutants compared to control embryos (blue) (*P*<0.01). In controls, internalization occurs 7±4 min (mean±s.d.) after the four-cell stage (*n*=14). Graphed results are mean±s.e.m. Student's *t*-test with Bonferroni correction was used for statistical analysis.

In addition to the ZF1 data suggesting a role for PI3K in phagocytosis, the P0 midbody is found on the outer surface of the embryo in *bec-1* and *vps-34* mutants (*n*=15/20 and *n*=8/11, respectively), showing it is not attached to a daughter cell. To confirm that PI3K is required for midbody phagocytosis, we looked for actin polymerization during midbody internalization. Cultured mammalian cells are known to internalize midbodies through an actin-dependent mechanism (Crowell et al., 2014). In addition, Beclin 1 acts upstream of RAC1 activation to promote actin rearrangements during apoptotic cell engulfment (Konishi et al., 2012). In control embryos, actin was enriched on the midbody shortly before internalization (Fig. 3A,B; Movie 2), likely driving the formation of a phagocytic cup. In contrast, actin fails to accumulate in *ced-2* engulfment mutants at the time of normal internalization (Fig. 3A,B; Movie 3). Actin is also not enriched around the midbody in *bec-1* mutants (Fig. 3A,B; Movie 4), allowing for the slower development of *bec-1* mutants (Fig. S2). This demonstrates that PI3K acts during midbody phagocytosis and that PI3K cannot be used to distinguish between phagocytic and autophagic degradation of the midbody.

**Fig. 4.**
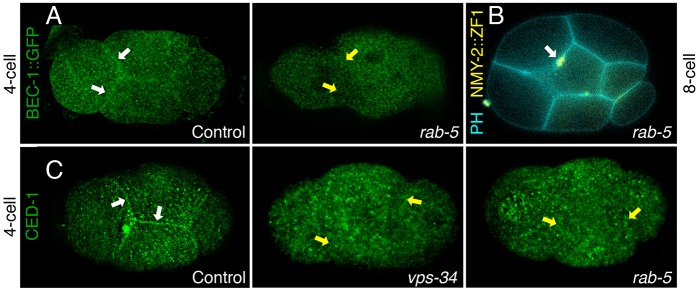
**RAB-5 and PI3K regulate trafficking of the phagocytic receptor CED-1 to the plasma membrane.** (A) BEC-1::GFP (green) localizes to the plasma membrane before midbody internalization (white arrows) in controls (left, *n*=22/25 embryos). Mild treatment with *rab-5* RNAi (right) prevents BEC-1::GFP localization to the plasma membrane (yellow arrows, *n*=14/15) and (B) prevents midbody internalization (*n*=15). NMY-2::GFP::ZF1 labels midbodies (yellow) and mCh::PH_PLCδ1_ labels cell membranes (PH, cyan). (C) CED-1 (green) localizes to the plasma membrane (white arrows) in control embryos (left, *n*=48/51), but fails to localize to the membrane (yellow arrows) in *vps-34* mutants (middle, *n*=10/14) or embryos treated with *rab-5* RNAi (right, *n*=63/68) and is only observed in cytoplasmic puncta. Antibody stainings were performed in triplicate.

To test how PI3K regulates midbody phagocytosis, we examined the localization of the PI3K subunit BEC-1. Staining for BEC-1::GFP shows weak cell cortex labeling (Fig. 4A, *n*=22/25), but it is not markedly enriched around midbodies at internalization. To test whether PI3K needs to localize to the plasma membrane during midbody internalization, we disturbed its localization. In other systems, the small GTPase Rab5 is required to localize PI3K to endosomes (Ravikumar et al., 2008). Partially depleting *rab-5* using a mild RNAi treatment prevented the cortical staining of BEC-1::GFP (Fig. 4A, *n*=14/15). Mild *rab-5* RNAi also disrupted midbody internalization (Fig. 4B; Table 1) similar to *vps-34* mutants (Fig. 2A), suggesting that PI3K is needed at the plasma membrane for midbody phagocytosis.

We next asked how PI3K regulates phagocytosis in *C. elegans*. Beclin-1-deficient mammalian cells show reduced recycling of phagocytic receptors to the plasma membrane due to a defect in recruiting the retromer complex (Lucin et al., 2013). In *C. elegans*, the phagocytic receptor CED-1 is recycled by the retromer complex to allow the clearance of apoptotic corpses (Chen et al., 2010). Thus, we predicted that PI3K regulates phagocytosis by recycling CED-1. Indeed, in contrast to control embryos, CED-1 does not localize to the plasma membrane in *vps-34* mutants (*n*=10/14) or in *rab-5* RNAi-treated embryos that lack cortical BEC-1 (*n*=63/68, Fig. 4C). Taken together, our data suggests the following sequence of events: RAB-5 regulates PI3K membrane localization, which in turn localizes the receptor CED-1 to the plasma membrane. CED-1 signaling is activated by the released midbody to activate the RAC1 homolog CED-10 to induce actin polymerization, driving phagocytosis (see model in Fig. 7).

### PI3K and Atg8/LC3 homologs are required for midbody phagosome maturation

**Fig. 5.**
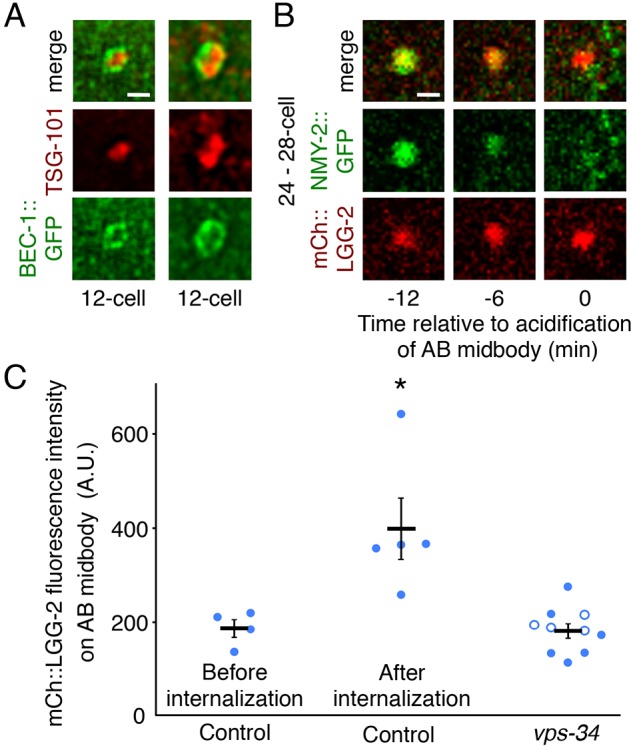
**Beclin 1 and LC3 homologs localize to the midbody phagosome.** (A) BEC-1::GFP (green) staining surrounds midbodies (TSG-101, red) after internalization (*n*=5/22 embryos). Antibody staining was performed in triplicate. (B) mCh::LGG-2 (red) localizes on midbodies (NMY-2::GFP, green, *n*=12/14) and persists after acidification. Scale bars: 1 μm (A,B). See also Fig. S3. (C) mCh::LGG-2 is significantly enriched on midbody phagosomes after internalization (*n*=4 before internalization, *n*=5 after internalization, *P<0.05 compared to midbodies before internalization). mCh::LGG-2 enrichment does not occur in *vps-34* mutants [*n*=6 released (solid blue circles), *n*=4 internalized midbodies (open blue circles)]. Results are mean±s.e.m. Student's *t*-test with Bonferroni correction was used for statistical analysis.

To determine why proteins classically associated with autophagy were required for phagosome degradation, we next analyzed the localization of PI3K and Atg8/LC3 proteins on phagocytosed midbodies. We found BEC-1::GFP staining enriched around internalized midbodies at the 12–15-cell stage (*n*=5/22 embryos, Fig. 5A). BEC-1 has been shown to act during the degradation of engulfed apoptotic bodies (Huang et al., 2013). Furthermore, VPS-34 has been shown to function in phagosome maturation (Kinchen et al., 2008), suggesting that PI3K could regulate the degradation of midbodies through phagosome maturation. We also observed that a mCh::LGG-2 reporter localized on internalized midbodies (*n*=12/14 embryos). The mCh:LGG-2 fluorescence was dimmer on midbodies than on autophagosomes in the one-cell embryo (Al Rawi et al., 2011), suggesting that the LGG-2-positive midbodies are not in autophagosomes. Atg8/LC3 proteins have been shown to localize on phagosomes during LC3-associated phagocytosis (LAP) in order to facilitate phagosome maturation and fusion with lysosomes (Sanjuan et al., 2007; Florey et al., 2011). Thus, both PI3K and Atg8/LC3 proteins localize to the midbody phagosome and could play a role in phagosome maturation.

We next examined the timing of LC3 accumulation on the midbody. Using live imaging, we found that mCh::LGG-2 was enriched on P0, AB and P1 midbodies after internalization (Fig. 5B,C; Fig. S3). LGG-1 depends on BEC-1 to localize to apoptotic phagosomes (Florey et al., 2011), suggesting that PI3K could help the midbody phagosome mature by acquiring Atg8/LC3-family proteins to facilitate fusion with lysosomes. We tested whether PI3K was required for LC3 enrichment by examining mCh::LGG-2 dynamics in *vps-34* mutants. We observed no enrichment of mCh::LGG-2 on midbodies in *vps-34* maternal-zygotic mutants, including on rare internalized midbodies (Fig. 5C). Thus, PI3K is required for the recruitment of LC3 to the midbody phagosome.

**Fig. 6.**
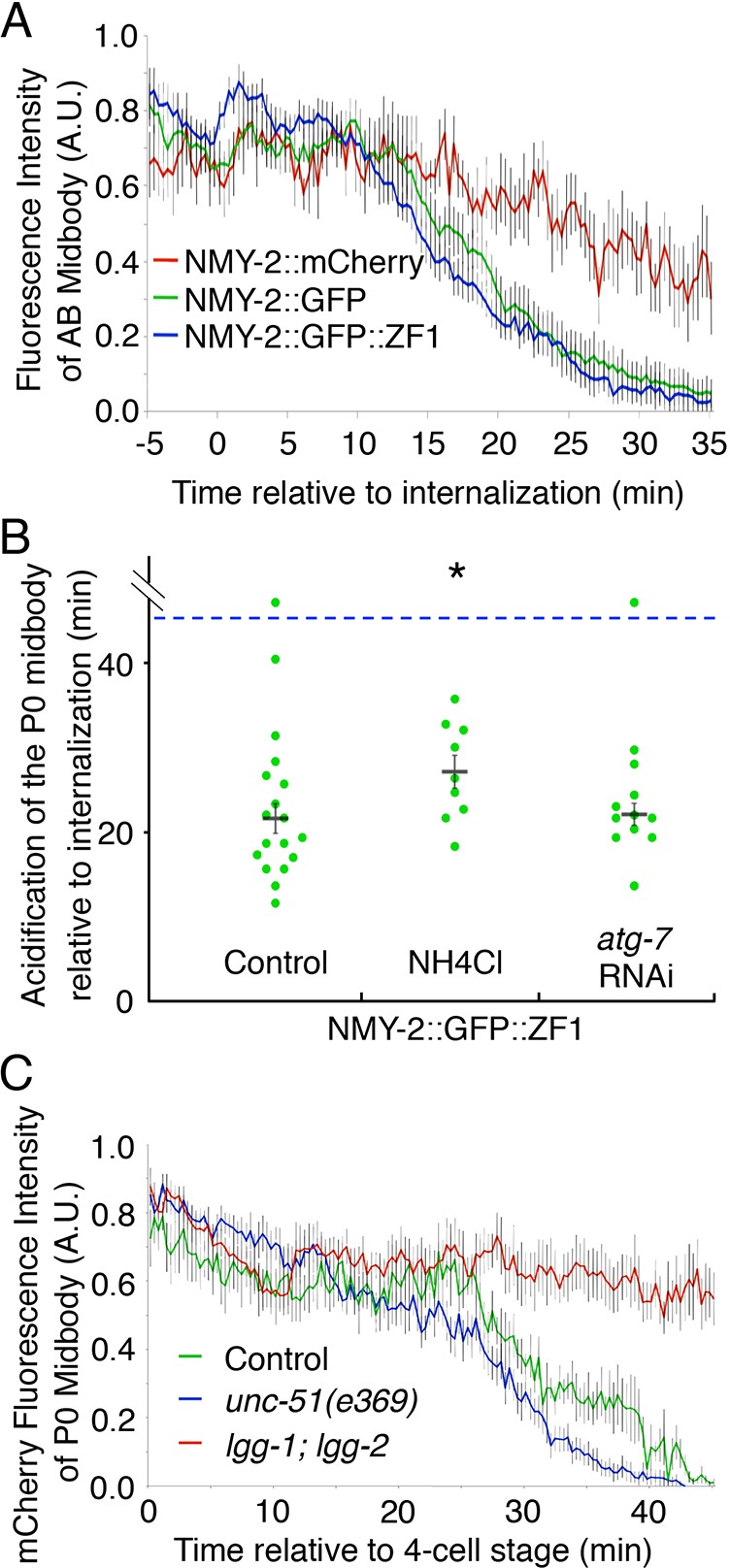
**Midbodies are degraded** **by** **LC3-associated phagocytosis.** (A) GFP-tagged NMY-2 with (blue, *n*=11 embryos) or without the ZF1 domain (green, *n*=7) starts to lose fluorescence by 15 min after AB midbody internalization (*P*<0.01), but mCherry-tagged NMY-2 (red, *n*=5) fluorescence persists longer. See also Fig. S4. (B) The disappearance of NMY-2::GFP::ZF1 was significantly delayed by NH_4_Cl treatment (*n*=9, *P*<0.05), but not by *atg-7* RNAi (*n*=12, *P*>0.05) compared to controls (*n*=18). (C) NMY-2::mCh fluorescence is more stable in the P0 midbody in *lgg-1; lgg-2* mutants (red, *n*=6) than in control WEH02 embryos (green, *n*=6) or *unc-51* mutants (blue, *n*=10) (*P*<0.01). Results are mean±s.e.m. Student's *t*-test with Bonferroni correction was used for statistical analysis.

While analyzing LC3 dynamics, we also observed that mCh::LGG-2 persisted on the midbody phagosome after NMY-2::GFP disappeared (Fig. 5B, *n*=2/5 embryos). This data suggested that disappearance of the GFP reporter could be due to phagosome acidification rather than midbody degradation, because GFP is known to be sensitive to pH. GFP(S65C) was used in all constructs, which is predicted to have a pKa of 6, similar to GFP(S65T) (Kneen et al., 1998; Green et al., 2008). In contrast, mCherry has a pKa<4.5 and thus maintains fluorescence at lower pH than GFP (Shaner et al., 2004). To ask what caused the disappearance of NMY-2::GFP (Fig. 5B) and NMY-2::GFP::ZF1 (Fig. 2B) after phagocytosis, we measured AB midbody fluorescence in strains expressing differently tagged NMY-2 reporters. NMY-2::GFP disappeared with similar timing (29±6 min after internalization, mean±s.d., *n*=7, Fig. 6A) to the ZF1-tagged reporter, revealing that the disappearance of NMY-2::GFP::ZF1 on internalized midbodies is not due to ZF1 degradation. In contrast to either GFP reporter, NMY-2::mCh sustained fluorescence significantly longer, disappearing 40±5 min after midbody internalization (*n*=4, Fig. 6A). Thus, the phagosome membrane protects midbodies from ZF1-mediated proteasomal degradation after internalization.

We confirmed this finding by testing whether other midbody components lose GFP and mCherry fluorescence with similar timing. The centralspindlin subunit ZEN-4 (KIF23 or MKLP1 in mammals) is an essential component of the motor complex at the spindle midzone and becomes associated with the midbody core (Green et al., 2013). ZEN-4::GFP disappeared with similar timing to NMY-2::GFP, whereas mCherry::ZEN-4 disappeared about 15 min later (Fig. S4). This difference in timing between GFP- and mCherry-tagged midbody reporters suggests that the loss of NMY-2::GFP, NMY-2::GFP::ZF1 and ZEN-4::GFP fluorescence is due to quenching of GFP fluorescence as the phagosome acidifies.

To confirm that loss of GFP fluorescence was due to acidification of the midbody phagosome, we inhibited acidification by treating worms with ammonium chloride. Ammonium chloride is an alkalizing agent known to prevent acidification of endosomes and lysosomes (Artal-Sanz et al., 2006). After treatment with NH_4_Cl, NMY-2::GFP::ZF1 fluorescence persisted significantly longer on midbodies than in untreated embryos (*P*<0.05, Fig. 6B). NH_4_Cl did not delay degradation of the cytoplasmic pool of NMY-2::GFP::ZF1 (*n*=9), demonstrating that NH_4_Cl did not disrupt ZF1 degradation. Thus, GFP disappearance after phagocytosis is due to phagosome acidification.

Because we had observed mCh::LGG-2 on the midbody prior to GFP disappearance (Fig. 5B), we asked whether autophagy proteins were required for phagosome acidification. We measured the disappearance time of GFP-labeled midbodies unable to lipidate Atg8/LC3 proteins. NMY-2::GFP::ZF1 disappeared with a similar timing to controls in embryos treated with *atg-7* RNAi (Fig. 6B), indicating that although LGG-2 associates with the midbody prior to acidification, lipidated LGG-1 and LGG-2 are not required for acidification of the midbody phagosome. Because a midbody left in the cytoplasm after a failed autophagic engulfment would not be expected to acidify, acidification in *atg-7* knockdowns is further evidence that macroautophagy does not sequester the midbody.

### Macroautophagy is not required for midbody degradation

Given that autophagy proteins are not required to sequester the midbody or acidify the phagosome, we asked whether macroautophagy was really involved in midbody degradation. We examined mutants in proteins that specifically affect macroautophagy and not LAP, namely the homologs of ULK1 and ATG14. ULK1 phosphorylates Beclin 1 to induce autophagy and ATG14 is an autophagy-specific regulator of PI3K, but neither protein is required for LAP (Martinez et al., 2011, 2015; Russell et al., 2013). In *C. elegans* embryos, the ULK1 homolog UNC-51 is required for the autophagy-dependent degradation of P granules, an unwrapped organelle, and paternal mitochondria, a double membrane-wrapped organelle. Using the *unc-51(e369)* mutant allele with defects in mitophagy (Sato and Sato, 2011), we discovered that NMY-2::mCh disappearance was not delayed on the P0 midbody (Fig. 1; Fig. 6C). Midbody internalization was also not disrupted in *unc-51(e369)* mutants (Table 1). Given that the premature stop codon in *e369* occurs after the kinase domain of UNC-51, we next examined the *unc-51*(*e1189)* allele, which severely reduces the amount of *unc-51* RNA (Ogura et al., 1994). In *unc-51(e1189)* mutants, P granules fail to be degraded by autophagy in the early *C. elegans* embryo (Zhang et al., 2009), but we found that midbody disappearance was not significantly altered (Fig. 1). These results demonstrate that macroautophagy initiated by UNC-51 is not required for midbody degradation.

To confirm our interpretation of the UNC-51 results, we next examined what happened to the midbody when EPG-8 was deleted. EPG-8 is the functional homolog of ATG14 and is required for the degradation of P granules by autophagy in *C. elegans* embryos (Yang and Zhang, 2011). The *epg-8(ok2561)* allele deletes both coiled coil domains necessary for EPG-8 binding to BEC-1, and *epg-8* mutant embryos fail to accumulate LGG-1-positive autophagosomes. Similar to *unc-51* mutants, NMY-2::mCh disappearance was not delayed in *epg-8* maternal-zygotic mutants (Fig. 1). Midbody internalization was also not disrupted in *epg-8* deletion mutants (Table 1). Therefore, EPG-8, a second autophagy-specific regulator, does not regulate midbody degradation. In summary, these data demonstrate that macroautophagy has no role in midbody internalization or degradation in *C. elegans* embryos. Thus, the data demonstrate that PI3K and Atg8/LC3 proteins act during LAP to mediate midbody phagosome maturation and degradation.

## DISCUSSION

### Model of midbody fate and signaling termination

**Fig. 7.**
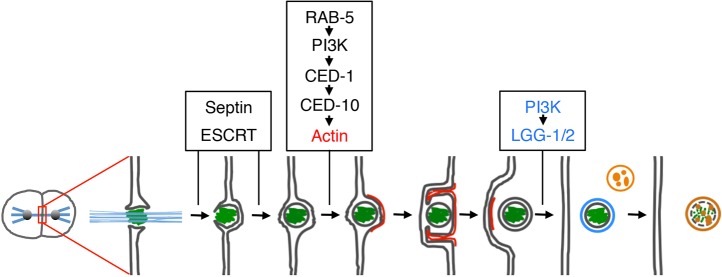
**Model of midbody fate.** The contractile ring closes the membrane (dark gray) to form the intercellular bridge containing microtubules (blue) and the midbody (green). Abscission starts on one side of the midbody and the remnant is released after cuts on both sides requiring the septin UNC-59 and the ESCRT-I component TSG-101. Actin enrichment (red) depending on RAB-5, PI3K, CED-1 and CED-10 drives phagocytic cup formation and the midbody is engulfed. The midbody phagosome matures through PI3K activity and acquires lipidated LGG-1 and LGG-2 (blue) before fusing with lysosomes (orange) for degradation. Each line represents a membrane bilayer.

In summary, by analyzing the role of both phagocytic and autophagic proteins in midbody internalization and degradation in *C. elegans*, we have developed a new model for midbody fate (Fig. 7). First, using a degradation technique to probe access to the midbody *in vivo*, we reveal that midbodies are released extracellularly and abscission occurs on both sides of the midbody, even in midbodies asymmetrically inherited by a daughter cell. Both single and double cuts have been observed around midbodies using electron microscopy (Elia et al., 2011; Guizetti et al., 2011), suggesting that abscission likely occurs asynchronously. In the context of a crowded tissue, released midbodies are then internalized by receptor-mediated phagocytosis driven by actin polymerization. Actin-dependent engulfment of released midbodies has also been observed in cultured cells using electron microscopy (Crowell et al., 2014). Extension of the phagocytic cup by actin polymerization requires the activation of the RAC1 homolog CED-10 through CED-1-receptor-mediated signaling from the plasma membrane (Chai et al., 2012). We show that CED-1 localization depends on the cortical activity of the class III PI3K complex and RAB-5, with CED-1 likely being recycled to the plasma membrane through retromer trafficking as seen with mammalian phagocytic receptors (Lucin et al., 2013). After phagocytosis, the outer membrane of the midbody phagosome matures using the PI3K complex to recruit LC3 and GABARAP homologs, which help the phagosome fuse with the lysosome for degradation of the midbody remnant. Thus, a conserved cascade of proteins regulates the membrane association of Atg8/LC3 proteins to serve similar roles on midbody phagosomes and autophagosomes.

That autophagy proteins are required for the degradation of midbody phagosomes might not be surprising considering that they are both double membrane structures that need to fuse with lysosomes. During macroautophagy, the phagophore provides a double membrane derived from the endoplasmic reticulum or other intracellular organelle (reviewed in Carlsson and Simonsen, 2015). During LAP, the outer phagosome membrane is derived from the plasma membrane and the inner membrane comes from the engulfed structure. In the case of the midbody, the inner membrane is derived from the plasma membrane wrapping the intercellular bridge. Cell corpses are also wrapped in their plasma membrane before phagocytosis (Florey et al., 2011; Martinez et al., 2011). Thus, in both autophagy and LAP, the cell must degrade the contents of a double membrane, which is facilitated by the incorporation of lipidated Atg8/LC3 proteins.

LAP was not well established when the role of macroautophagy in midbody degradation was proposed. The requirement for proteins like PI3K and LC3 was attributed to autophagic sequestration (Pohl and Jentsch, 2009; Kuo et al., 2011), despite the lack of evidence for unwrapped midbodies in the cytoplasm. Similarly, electron microscopy studies demonstrating that mammalian midbodies are wrapped in a double membrane are equally consistent with release followed by LAP and macroautophagy of cytoplasmic midbodies (Ettinger et al., 2011). To date, no one has tested a role for ULK1 or ATG14 in midbody degradation in mammals, which could distinguish between autophagy and LAP. p62 (also known as SQSTM1), NBR1, WDFY3 and TRAF6 act early in ubiquitin-dependent autophagy and are required for midbody degradation (Pohl and Jentsch, 2009; Kuo et al., 2011; Isakson et al., 2013), but it is untested whether these proteins are also used during LAP. In addition, studies showing that different mammalian cell types release midbodies at different rates could be caused by differences in midbody remnant adhesion or phagocytosis, rather than differences in midbody release (Ettinger et al., 2011; Crowell et al., 2014). Thus, the existing data from mammalian cells does not distinguish macroautophagy from LAP and the published evidence for macroautophagy could equally be interpreted as evidence for LAP in the degradation of mammalian midbodies. In summary, although we cannot exclude that macroautophagy is sometimes used to degrade cytoplasmic midbodies in other systems, we predict that midbody release followed by LAP is the primary *in vivo* fate of internalized midbodies in animals.

The midbody provides the first example for the use of LAP to degrade endogenous organelles. In addition to LAP-mediated degradation of pathogens and cell corpses (Sanjuan et al., 2007; Florey et al., 2011), LAP is also important for vision and is used by the retinal pigment epithelium to degrade shed photoreceptor outer segments (Kim et al., 2013). Recently, LAP has been shown to protect mice from lupus, indicating that the role of autophagy proteins in inflammatory responses during autoimmune disease is due to their role in LAP (Martinez et al., 2016). Our findings also suggest that LAP is part of the normal physiology of a cell and is used to protect the cell from dangerous cargos over a large size range, even when self-derived. Releasing the midbody after cytokinesis ensures that cytoplasmic midbody proteins will be unable to continue to signal to the cytoskeleton or regulate vesicular trafficking. Internalizing the midbody remnant in a protective membrane also terminates signaling from lipids and proteins on the midbody surface in a crowded tissue environment. Post-mitotic midbody positioning has been shown to influence cell polarity and cell fate (Kuo et al., 2011; Morais-de-Sa and Sunkel, 2013; Singh and Pohl, 2014) and the released midbody is able to engage multiple pathways of receptor-mediated signaling to induce phagocytosis (Chai et al., 2012; Ou et al., 2014). Thus, cells carry out a complex series of membrane remodeling events to ensure that midbody signaling is promptly terminated.

## MATERIALS AND METHODS

### Worm strains and maintenance

*Caenorhabditis elegans* strains were maintained according to standard protocol (Brenner, 1974). For a list of strains used in this study, see Table S1. To generate *unc-59* loss-of-function mutant embryos, the WEH132 strain with a hypomorphic *unc-59* mutation was treated with *unc-59* RNAi. To generate *lgg-1; lgg-2* maternal-zygotic mutant embryos, the lethality of *lgg-1; lgg-2* mutants was rescued with an *lgg-1::gfp* transgene that is not visibly expressed in the early embryo, similar to in Manil-Segalen et al. (2014). To further deplete maternal protein, L3 worms were treated with *lgg-1* RNAi. The absence of LGG-1::GFP transgene expression was verified by staining with an anti-GFP antibody. To generate *bec-1* maternal-zygotic mutant embryos, embryos were isolated from rare fertile *bec-1* homozygotes (<5%), whereas *vps-34* maternal-zygotic mutant embryos were isolated as described previously (Lu et al., 2012). Embryos were scored after time-lapse imaging for the presence of the rescuing *vps-34* extrachromosomal array. Weak or occasional maternal transcription from the rescuing array is likely responsible for the weaker phenotypes of *vps-34* mutants compared to *bec-1* mutants (Table 1). To generate *epg-8* maternal-zygotic mutant embryos, embryos were isolated from *epg-8* homozygotes.

### Worm transformation

WEH06, WEH45 and WEH59 strains were made by biolistic transformation (Bio-Rad PDS-1000, Munich, Germany) of the HT1593 strain as described previously (Praitis et al., 2001) with the following GFP-tagged fosmids from the TransgenOme project (Dresden, Germany) (Sarov et al., 2012): WRM0626D_F06(pRedFlp-Hgr)(bec-1[23858]::S0001_pR6K_Amp_ 2xTY1ce_EGFP_FRT_rpsl_neo_FRT_3xFlag)dFRT::unc-119-Nat; WRM068A_G10(pRedFlp-Hgr)(lgg-1[30711]::S0001_pR6K_Amp_ 2xTY1ce_EGFP_FRT_rpsl_neo_FRT_3xFlag)dFRT::unc-119-Nat; and WRM0636D_G11(pRedFlp-Hgr)(zen-4[33383]::S0001_pR6K_Amp_2xTY1ce_EGFP_FRT_rpsl_neo_FRT_3xFlag)dFRT::unc-119-Nat.

### RNAi experiments

RNAi was performed by feeding double-stranded RNA (dsRNA)-expressing bacteria from the L1 larval stage through adulthood at 25°C (60–70 h) according to established protocols (Fraser et al., 2000). For *lgg-1* RNAi, worms were treated starting at the L3 stage to avoid larval arrest (Manil-Segalen et al., 2014). For *rab-5* RNAi, WEH06 worms were treated starting at the L3 stage to avoid larval arrest and sterility (Kinchen et al., 2008; Green et al., 2011) or L1 stage feeding was performed in the WEH141 strain bearing a *rrf-1* mutation to cause ‘germ-line-only RNAi’ (Sijen et al., 2001). These partial RNAi treatments are likely responsible for the milder phenotypes observed in embryos treated with *rab-5* RNAi compared to *bec-1* and *vps-34* maternal-zygotic mutants (Table 1). RNAi constructs were obtained from available libraries (Source BioScience). The following clones were used: *atg-7* (sjj_M7.5), *ced-10* (mv_C09G12.8), *lgg-1* (mv_C32D5.9), *rab-5* (mv_F26H9.6) and *unc-59* (mv_W09C5.2). 0.5-2 µg/µl *tsg-101* and 0.5 µg/µl *zif-1* dsRNA were injected into the gonad of young adult worms 20–26 h before analysis. The dsRNA was transcribed using T7 RNA Polymerase (ThermoFisher) from T7 PCRs from cDNA cloned into the RNAi plasmid L4440 (Kamath and Ahringer, 2003). The *tsg-101* dsRNA was designed according to the Cenix clone 49-f7 (Sonnichsen et al., 2005). Efficiency of *tsg-101* RNAi was judged by a mild delay in internalization of the AB midbody; embryos that internalized the AB midbody at the six-cell stage were excluded from ZF1 degradation analysis. The *zif-1* dsRNA was designed using the following primers: forward, 5′-CGTCAAGAACATCAGAGCATAGCA-3′; and reverse, 5′-CGGCAGTTTCTTTTTCAAGTTCTTC-3′.

### Ammonium chloride treatment

L4 worms were picked to a watch glass containing M9 buffer supplemented with OP50 bacteria and 10 μM NH_4_Cl and were shaken for 15–20 h. Longer NH_4_Cl treatment delayed embryonic development.

### Time-lapse imaging

Embryos were dissected from gravid adults and mounted in M9 buffer on an agarose pad on a slide, except embryos from NH_4_Cl-treated worms were mounted in M9 buffer containing 40 μm NH_4_Cl. Z-stacks were acquired sequentially for GFP and mCherry every 20 s at room temperature using a Leica DM5500 wide-field fluorescence microscope with a HC PL APO 40×1.3 NA oil objective lens supplemented with a Leica DFC365 FX CCD camera controlled by LAS AF software. The live colocalization images in Fig. 5B and Fig. S3 were obtained simultaneously using a Leica TCS SP5 confocal microscope with a HCX PL APO 63×1.4 NA oil objective lens supplemented with a Leica HyD hybrid detector. Time-lapse series were analyzed using Imaris (Bitplane). The four-cell stage is defined as the beginning of P1 furrow ingression. Internalization is defined as the first frame where the midbody moves away from the plasma membrane, which is likely to closely reflect closure of the phagocytic cup because it correlates with bright actin accumulation.

### Image manipulation

For clarity, images were rotated, colorized to cyan and yellow, and the intensity was adjusted using Adobe Photoshop. In Figs 3A,C and 5A, the *z*-stack was first deconvolved using LAS AF software. Only one *z*-plane is shown except for Fig. 5A,B and Fig. S3, where 2 *z*-planes are projected (z interval of 0.25, 0.3 μm and 0.3 μm, respectively). Videos were rotated and colorized, and the intensity adjusted using Imaris.

### Fluorescence intensity measurement

Mean fluorescence intensity was measured in a circle with area of 0.5 μm^2^ for NMY-2 using ImageJ (NIH). For LifeAct::RFP measurements, a 2-µm circle was used. For mCherry::LGG-2 intensity, a circle of between 0.8 and 1.1 μm^2^ was used. Midbody fluorescence was measured from contractile ring closure until the end of the movie or until the midbody was not distinguishable from the cytoplasm. Fluorescence intensity of the first polar body was measured as an internal control. An exponential decay curve was fitted to the polar body data using OriginPro (OriginLab) and used to correct for fluorescence loss due to photobleaching. Embryos were excluded if the P0 and AB midbodies were too close to each other or if the polar body data did not fit an exponential decay function. NMY-2 data are reported as the ratio of the fluorescence intensity of the midbody to the expected value of the polar body after cytoplasmic background subtraction. For LifeAct measurements, normalization was performed using the fluorescence intensity of the cytoplasm. For mCherry::LGG-2 measurements, the ratio of the fluorescence intensity on the AB midbody to the neighboring cytoplasm is reported. Pre-internalization was measured at the brightest time point between the four- and six-cell stages in control embryos. Post-internalization, the brightest time point between the eight- and 24-cell stages was measured in both control embryos and *vps-34* mutants.

### Antibody staining

Gravid worms were dissected in water on a coverslip to release embryos and transferred to 0.1% polylysine-coated slides followed by immediate freezing on dry ice. The eggshell was cracked by flicking off the coverslip and the embryos were fixed in methanol before staining with the following antibodies: chicken anti-GFP 1:500 (cat. no GFP-1010, Aves, Tigard, OR), rabbit anti-TSG-101 1:1000 (gift of Jon Audhya, Madison, WI), and mouse anti-CED-1 1:500 (gift of Chonglin Yang, Beijing, China). Before staining for BEC-1::GFP with anti-GFP antibody, slides were also fixed with 4% formaldehyde solution (Anderson et al., 2008). BEC-1::GFP membrane localization was scored blind from randomized stained images of four-cell control and *rab-5*-RNAi-treated WEH06 embryos. CED-1 membrane localization was scored from two- to eight-cell stage embryos from control and *rab-5*-RNAi-treated N2 strains as well as the *vps-34* mutant strain ZH1057. Not all embryos from ZH1057 are *vps-34* maternal-zygotic mutants, because 50% contain a rescuing array that drives zygotic transcription of *vps-34* (*n*=36). The array likely leads to occasional weak maternal expression. Therefore, the requirement for PI3K in CED-1 (*n*=10/14) localization is likely underestimated by analyzing embryos from *vps-34* mutants carrying a rescuing array.

### Cell cycle timing

To compare the speed of development between control and mutant embryos, the time from the six- to the 12-cell stage (one cell cycle in the AB lineage) and the time from the four- to the 14-cell stage (two subsequent cell cycles in the EMS lineage) were calculated from time-lapse series. The cell cycle time was measured from the ingression of the furrow in ABx cells or the P1 cell to the ingression of the furrow in ABxx cells or MSx cells. For Fig. S2, the beginning of each stage was defined by furrow ingression and the P1 furrow ingression time was set as time zero.

### Statistical evaluation

Student's one-tailed *t*-test was used to test statistical significance using the Bonferroni correction to adjust for multiple comparisons. Mean±s.e.m. is depicted in graphs. Mean±s.d. is reported in the text and in Fig. S2.
